# SARS-CoV-2 genomic surveillance using self-collected saliva specimens during occupational testing programs

**DOI:** 10.3389/fpubh.2025.1360862

**Published:** 2025-04-08

**Authors:** Andrew T. Schnaubelt, David M. Brett-Major, Janet Williamson, Bailey Barcal, Julie Carstens, Ashley Peer, Michael Wiley, M. Jana Broadhurst

**Affiliations:** ^1^Department of Pathology, Microbiology, and Immunology, College of Medicine, University of Nebraska Medical Center, Omaha, NE, United States; ^2^Department of Epidemiology, College of Public Health, University of Nebraska Medical Center, Omaha, NE, United States

**Keywords:** SARS-CoV-2, COVID-19, saliva, genome sequencing, variant, surveillance

## Abstract

Continually emerging SARS-CoV-2 variants pose challenges to clinical and public health interventions, necessitating sustainable approaches to real-time variant monitoring. This case study describes an innovative SARS-CoV-2 screening and surveillance program that demonstrates the utility of sequencing-based variant monitoring using self-collected saliva specimens. We conducted saliva-based SARS-CoV-2 screening in occupational settings in Omaha, Nebraska from December 2021 through November 2022. 8,372 saliva specimens collected from 1,480 participants were tested for SARS-CoV-2 RNA by extraction-free PCR, with 334 positive samples referred for whole-genome sequencing analysis. Program utilization, quality metrics, and sequencing outputs were compared across sites. Specimen quality was high across program settings, demonstrating the suitability of self-collected saliva specimens for PCR and genomic surveillance testing. Virus RNA sequencing successfully determined the variant strain in 83 and 67% of SARS-CoV-2-positive saliva samples collected in two program settings, demonstrating the successful integration of SARS-CoV-2 sequencing for variant determination into screening programs in occupational settings using self-collected saliva with an extraction-free qRT-PCR testing method. We further demonstrate that the sensitivity and efficiency of variant analysis is dependent on the PCR cycle threshold (Ct) cutoff of the diagnostic assay virus gene target. Use of an optimized Ct value cutoff for sequencing referral is recommended. Community-based saliva testing programs can be utilized to enhance variant monitoring, and could be considered in the risk identification of other respiratory infections. This approach offers the advantages of a non-invasive specimen collection, no need for supervised collection by a healthcare worker, supply chain resiliency, distributable access, and scalability.

## 1 Introduction

SARS-CoV-2 variants of concern continue to emerge globally (1–3) and surveillance testing to detect and monitor variant strains remains a public health priority (4, 5). Surveillance testing for SARS-CoV-2 variant strains primarily relies on virus whole-genome sequencing, typically performed on nasal or nasopharyngeal swab specimens referred to clinical or public health laboratories for polymerase chain reaction (PCR)-based diagnostic testing. Gaps in access to and/or utilization of PCR-based diagnostic testing for SARS-CoV-2 pose a risk to adequate representation of underserved communities in genomic surveillance data. There is a critical need to develop and evaluate community-based testing strategies that increase the representation of high-risk populations in SARS-CoV-2 screening and genomic surveillance programs.

Community-based testing programs increase testing access through facilitated specimen collection in locations such as schools and workplaces, offering an important opportunity to expand representation for SARS-CoV-2 variant surveillance. Saliva has been increasingly utilized for such programs due to the ease of collection, greater acceptability due to a less invasive sampling method compared to nasopharyngeal swabs, specimen stability, and diagnostic reliability (6–9). Genomic sequencing of SARS-CoV-2 from saliva specimens has shown comparable results to nasopharyngeal swabs with specimens collected in a healthcare setting (10). However, the performance of integrated SARS-CoV-2 genomic sequencing during community-based saliva testing programs has not been described. Viral load distribution and specimen quality are major factors impacting the utility of sequencing strategies.

In Fall 2020, we developed and implemented an extraction-free PCR test for SARS-CoV-2 diagnosis using self-collected saliva to address regional gaps in testing access and to meet the need for community-based testing in Nebraska (11). During subsequent waves of the COVID-19 pandemic driven by variant strains of the virus, we developed an innovative community-based testing approach using self-collected saliva specimens for SARS-CoV-2 detection and variant determination by whole-genome sequencing to enhance public health surveillance of emerging SARS-CoV-2 variants. Here we describe program utilization, quality metrics, and outcomes of SARS-CoV-2 genomic sequencing performed on saliva specimens generated from occupational settings in Omaha, Nebraska and tested at the University of Nebraska Medical Center (UNMC). Genomic sequencing is resource intensive and warrants a strategic approach to optimize specimen referral. We examine the sensitivity and efficiency of genomic sequencing for variant analysis in the context of a specimen selection strategy based on the PCR cycle threshold (Ct) value as a measure of viral RNA quantity. This community case study describes an innovative approach to SARS-CoV-2 screening and genomic surveillance, and presents a data-driven strategy to monitor specimen quality and optimize specimen referral for sequencing, key factors in overall program efficacy. This work demonstrates the utility of self-collected saliva to expand SARS-CoV-2 genomic surveillance for variant monitoring enabling successful program implementation and expanded surveillance in high-risk occupational settings.

## 2 Methods

### 2.1 Program settings

The specimens and testing included in this analysis are from saliva-based SARS-CoV-2 diagnostic testing programs supported by the UNMC Emerging Pathogens Laboratory in two program settings between December 2021 and November 2022: (1) *UNMC campus (Omaha, Nebraska).* Voluntary saliva testing was made available to employees and students four days per week. Specimens were self-collected at an on-campus collection site with supervision by trained staff and hand-carried to the laboratory. (2) *Omaha workplaces (various locations in Omaha, Nebraska).* Mandatory employee saliva testing occurred prior to aggregated work activities, according to industry and organizational requirements. Specimens were self-collected at home or at temporary workplace collection sites with supervision by trained community members and delivered to the laboratory by ground couriers. All individuals who registered for these testing programs and submitted at least one specimen for testing were included in this study. This study received a non-human subjects research determination from the University of Nebraska Medical Center Institutional Review Board, as the work constituted quality assurance within a public health surveillance activity.

### 2.2 Program site preparation and training, test ordering and reporting

Each site managed their own saliva collection schedules and programs, with training and supplies provided by the testing laboratory. SARS-CoV-2 testing was provided at no cost to the individuals who sought testing. Representatives from each program site were trained on test ordering, specimen collection and packaging, and specimen transport or shipping to the laboratory. Registration and consent for saliva testing and electronic reporting was provided through the web-based Nebraska University Laboratory Information Reporting Tool (NULirt) system (12). NULirt reports individual test results by email. NULirt also provides electronic results reporting to the Department of Health and Human Services disease surveillance system, Nebraska electronic disease surveillance system, and the Centers for Disease Control and Prevention health information exchange.

### 2.3 Saliva specimen collection

Saliva self-collection was performed by the passive drool method (13), using the clinically validated Nebraska COVID-19 Saliva PCR Test Saliva Collection Kit under the supervision of trained staff or volunteers, or unsupervised using the Nebraska COVID-19 Saliva PCR Test Home Collection Kit. Half-length plastic straws (S.P. Richards Co) were used to collect saliva into dry 1.5 mL collection tubes (Eppendorf Co). Following surface decontamination with ethanol, each sample was placed in an individual biohazard bag and then packaged as a batch into a transport or shipping box. The packaged saliva specimens were transported or shipped under ambient conditions in accordance with International Air Transport Association regulations. Packaged SARS-CoV-2-positive saliva specimens were tested for the stability of RNA target detection following up to 96 h of exposure to summer or winter shipping conditions in accordance with FDA guidance (14). No significant drift in cycle threshold (Ct) values for the SARS-CoV-2 RNA target or the human RNA internal control target were observed in the stability study.

### 2.4 Saliva SARS-CoV-2 PCR testing and sequencing analysis

SARS-CoV-2 detection in saliva by quantitative reverse-transcription (qRT)-PCR was conducted by the CLIA-certified EPL at UNMC using the Nebraska COVID-19 Saliva PCR Test, as previously described (11). This diagnostic test is a dual-plex, extraction-free qRT-PCR assay adapted from the SalivaDirect protocol (15, 16) that detects SARS-CoV-2 N1gene RNA in saliva. In brief, 50 μL of well-mixed saliva is added to 6.3 μL of proteinase K (New England Biolabs), then shaken and heated at 2,200 RPM and 95°C for 5 min. Five microliters of the sample preparation are added to 15 μL of PCR master mix (TaqPath 1-Step RT-1PCR Master Mix, ThermoFisher Scientific) containing primers and probes for detection of SARS-CoV-2 N1 gene RNA and human ribonuclease P (RNase P) RNA (Integrated DNA Technologies). RT-PCR is performed on QuantStudio7 pro thermocyclers (ThermoFisher Scientific). A positive test is defined by N1 gene target amplification with a Ct value <40; higher levels of gene target yield lower Ct values. Human RNase P RNA is measured as an internal control for specimen adequacy and amplification efficiency. A test was considered valid if the RNase P gene target amplification was adequate with a Ct value <35.

SARS-CoV-2 sequencing supported a national initiative funded by the Centers for Disease Control and Prevention to expand surveillance for emerging variant strains. Saliva specimens that tested positive for the presence of SARS-CoV-2 were stored frozen at −20°C until they were processed for genomic sequencing. Nucleic acid was extracted from 400 μL of saliva using the MagMAX™ Viral/Pathogen II (MVP II) Nucleic Acid Isolation Kit (Applied Biosystems, A48383) on the KingFisher Flex instrument following the manufacturer’s protocol and resuspended in 55 μL of elution buffer. Both Illumina and Oxford Nanopore Technology (ONT) sequencing platforms were used for supply chain resiliency. RNA was reverse transcribed using the SuperScript™ IV First-Strand Synthesis System (ThermoFisher, 1,809,150). For the Illumina based sequencing, SARS-CoV-2 was amplified and converted to sequencing libraries with the xGen SARS-CoV-2 Amplicon Panel (IDT,10009827, 10,009,832, 10,009,845). Libraries were normalized to 4 nM using Normalase and loaded on a 2 × 150 MiniSeq High Output cartridge. Consensus genomes were generated using the TAYLOR pipeline with default settings (17, 18). For the ONT based sequencing, SARS-CoV-2 was amplified and libraries were prepared according to the nCoV-2019 sequencing protocol (GunIT) v2 developed by the ARTIC Network (19), and loaded onto a R9.4.1 MinION flow cell. Consensus genomes were generated using the ARTIC network pipeline with default settings (20). SARS-CoV-2 consensus genomes with at least 70% coverage were submitted to Nextclade (21) for variant identification.

### 2.5 Program performance monitoring and evaluation

Program performance was assessed using both process and outcome measures. The diagnostic testing process measures were RNase P Ct value results and turnaround time. The RNase P internal control result represents specimen adequacy. The turnaround time uses the time it takes from specimen collection to results reporting as a surrogate for consistency of the overall process. The outcome measures were sample rejection rate and invalid result rate. The sample rejection rate reflects the proportion of samples that were not able to be processed due to inadequate labeling, insufficient volume, container leakage, receipt in the lab >96 h from collection, or other issues that impact specimen integrity. The invalid result rate reflects the proportion of samples that yielded inconclusive or unreliable results. These measures reflect diagnostic yield returned to both participants and health systems. The process measure for SARS-CoV-2 genomic sequencing was the number of specimens which yielded sequence coverage greater than 70%. The outcome measure was the percentage of specimens that yielded a variant determination that was reportable to public health authorities. Performance measures are reported for two program settings.

N1 Ct values were assessed in two ways to evaluate the relationship between diagnostic testing results and subsequent referral to sequencing activities. First, they were rank-ordered by sensitivity to determine how many of the achieved variant calls would have been captured if the Ct value was used as a threshold for referral to sequencing (sensitivity rank score). Second, they were rank-ordered by efficiency to determine the fraction of referred specimens that would have yielded a variant determination if that Ct value was employed as the threshold for referral (efficiency rank score). For each Ct value threshold, the sensitivity rank score was then multiplied by the efficiency rank score to provide an overall rank score.

### 2.6 Demographic, test utilization, and statistical analyses

Demographic information and test utilization metrics were extracted from test registration and required public health reporting data elements in the NULirt information reporting tool. Comparative data for Douglas County, Nebraska (resident county of Omaha) was downloaded from the CDC COVID-19 Data Tracker (22).

Data management and descriptive analytics were conducted in Microsoft Excel ® and SAS Version 9.4®. Visualization of testing, positive results, and case counts was accomplished with GraphPad Prism 9.5.1.

## 3 Results

### 3.1 Utilization of saliva SARS-CoV-2 testing in occupation settings in Omaha, Nebraska

**Figure 1:**
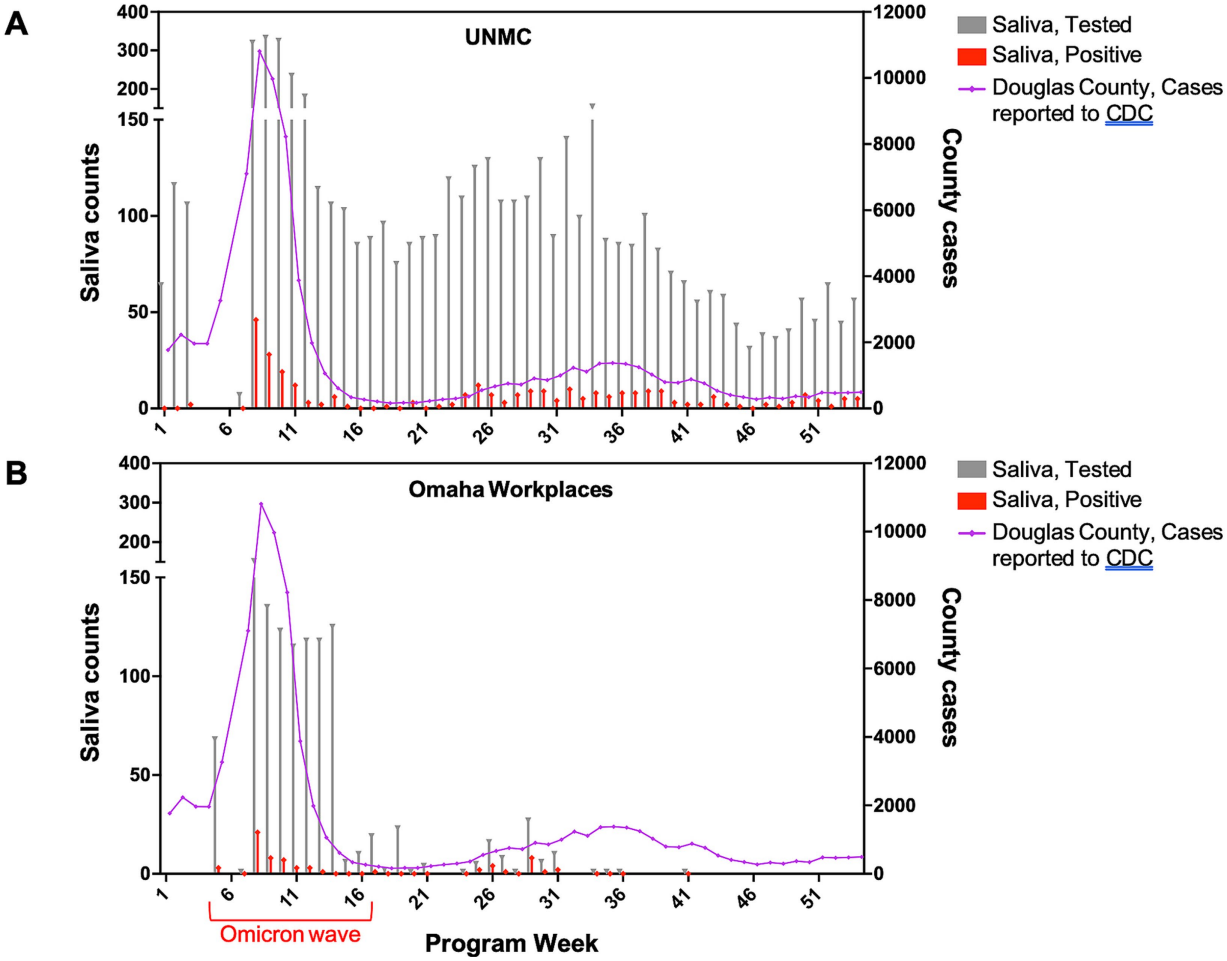
Weekly saliva tests performed and positive tests by program setting, December 2021–November 2022.

Demographic and individual-level test utilization metrics are provided in Table 1. Figure 1 shows the weekly counts of tests performed and positive test results for both program settings over the study period. At UNMC, the availability of unrestricted, voluntary testing four days per week resulted in a higher number of tests performed and more repeated testing among individual participants compared to participants in other occupational settings (Table 1). Test utilization rates at UNMC peaked during the community case surge related to the emergence of the Omicron variant (Figure 1A). Mandatory screening testing in workplace settings in Omaha also peaked during the surge in community cases related to the emergence of the Omicron variant (Figure 1B) while a sharp reduction in test utilization occurred as organizations de-escalated COVID-19 risk mitigation requirements. In general, as community transmission of COVID-19 increased, more individuals participated in saliva testing programs and a higher proportion of those individuals tested positive for the virus. At UNMC, women and those of Hispanic ethnicity appeared to have lower test positivity rates than men or those not self-identifying as Hispanic (Table 1).

**Table 1:** Participant demographics and test utilization by program setting, December 2021–November 2022.

	UNMC campus		Omaha workplaces	
	Ever +^a^	Never +^b^	Ever +	Never +
*n (%)*	232^c^ (20)	930 (80)	64 (20)	254 (80)
*Age*				
Mean years (SD; range)	39 (14; 18–79)	37 (14; 6–80)	45 (13; 22–70)	45 (14; 9–79)
*Gender*				
(Women %)	118 (51)	596 (64)	21 (33)	81 (32)
*Race (%)*				
African American	13 (6)	37 (4)	12 (19)	35 (14)
American Indian or Alaska Native	*	*	0	*
Asian	53 (23)	171 (18)	0	*
Native Hawaiian or Other Pacific Islander	0	*	0	0
White	150 (65)	622 (67)	49 (77)	202 (80)
Other	*	*	*	*
*Hispanic ethnicity (%)*	21 (9)	136 (15)	3 (5)	15 (6)
*No. of tests per individual*				
Mean ^d^ (SD; range)	16 (25; 1–149)	9 (12; 1–75)	3 (2; 1–10)	3 (2; 1–13)
*Interval between tests (days)*				
Mean (SD; range)	20 (38; 0–294)	21 (39; 0–309)	15 (26; 0–123)	10 (16; 0–156)

^a^Ever + is defined as a participant with a positive SARS-CoV-2 RT-PCR test result at any time during the program evaluation period.

^b^Never + is defined as a participant never having a positive SARS-CoV-2 RT-PCR test result at any time during the program evaluation period.

^c The UNMC program setting had 2 individuals with 2 positive results > 30 days apart. These individuals were included twice as Ever +.^.

^d^ This mean was calculated as the mean serial of tests within each class (e.g., UNMC ever positives) so reflects the population distribution of tests (comparative skew) rather than being an aggregate of number of times testing was used by each individual.

*The demographic was present but constituted < 5% of the population in its category. In each case, the distribution was comparable between Ever + and Never +.

### 3.2 Quality metrics for saliva SARS-CoV-2 PCR testing and genomic sequencing

Self-collected saliva specimens demonstrated reliable quality for PCR testing in both program settings, despite variable collection venues, specimen transport times, and environmental conditions over four seasons. Low rates of specimen rejection (<0.3%) and invalid results (<0.2%) were observed (Table 2). The time from specimen collection to receipt in the laboratory varied between program settings (average 3.4 and 11.4 h for UNMC and Omaha workplaces, respectively; Table 2). However, Ct values for the RNase P internal control gene target were comparable for specimen sets from both program sites (average 22.0 and 21.7 for UNMC and Omaha workplaces, respectively; Table 2) indicating stability of RNA for PCR analysis.

**Table 2:** Quality metrics for saliva SARS-CoV-2 PCR testing and genomic sequencing by program setting, December 2021–November 2022.

Quality Metrics		UNMC campus	Omaha workplaces
		*n* = 5,303 specimens received	*n* = 3,069 specimens received
Specimen quality	No. Rejected (%)	3 (0.06)	8 (0.27)
	No. Invalid (%)	5 (0.10)	4 (0.13)
	Av. RNase P Ct (SD)	22.0 (2.7)	21.7 (2.4)
Turnaround time (hrs)	Av. Collected-received	3.4	11.4
	Av. Received-reported	2.8	3.2
		*n* = 262 SARS-CoV-2+ with sequencing attempted^a^	*n* = 72 SARS-CoV-2+ with sequencing attempted
Genomic sequencing	Av. N1 Ct (SD; range)	28.3 (5.6; 13.7–38.2)	27.5 (6.3; 10.7–39.9)
	No. with variant determined (%)	217 (83)	48 (67)
	No. with genome coverage ≥99% (%)	103 (39)	9 (13)

^aNumber of specimens with sequencing attempted may exceed the number of PCR-positive participants due to occurrences of serial PCR-positive specimens.^

SARS-CoV-2 whole-genome sequencing was attempted for all PCR-positive saliva specimens when resources permitted. During a reagent shortage in January 2022 (coinciding with a surge in cases due to the emergence of the Omicron variant) specimens with N1 Ct values >32 were de-prioritized for sequencing referral, resulting in the exclusion of 8/342 (2.3%) PCR-positive specimens from sequencing analysis. N1 gene target Ct values in PCR-positive specimens referred for sequencing showed similar averages (28.3 and 27.5 for UNMC and Omaha workplaces, respectively; Table 2) and distributions (Supplementary Figure S1) in both program settings, suggesting comparable viral load experiences in the participant populations. SARS-CoV-2 variant determination (requiring at least 70% genomic sequence coverage) was achieved in 217/262 (83%) and 48/72 (67%) of PCR-positive saliva specimens collected from UNMC and Omaha workplaces, respectively (Table 2). Variants detected across program periods included B.1.617.2, B.1.1.529, BA.1 and sublineages, BA.2 and sublineages, BA. 4 and sublineages, and BA.5 and sublineages (Supplementary Figure S2). SARS-CoV-2 sequence coverage of ≥99% (i.e., adequate for phylogenetic analysis for outbreak investigation) was achieved in fewer specimens (Table 2).

### 3.3 Examination of optimal Ct value to trigger referral for sequencing analysis

**Figure 2:**
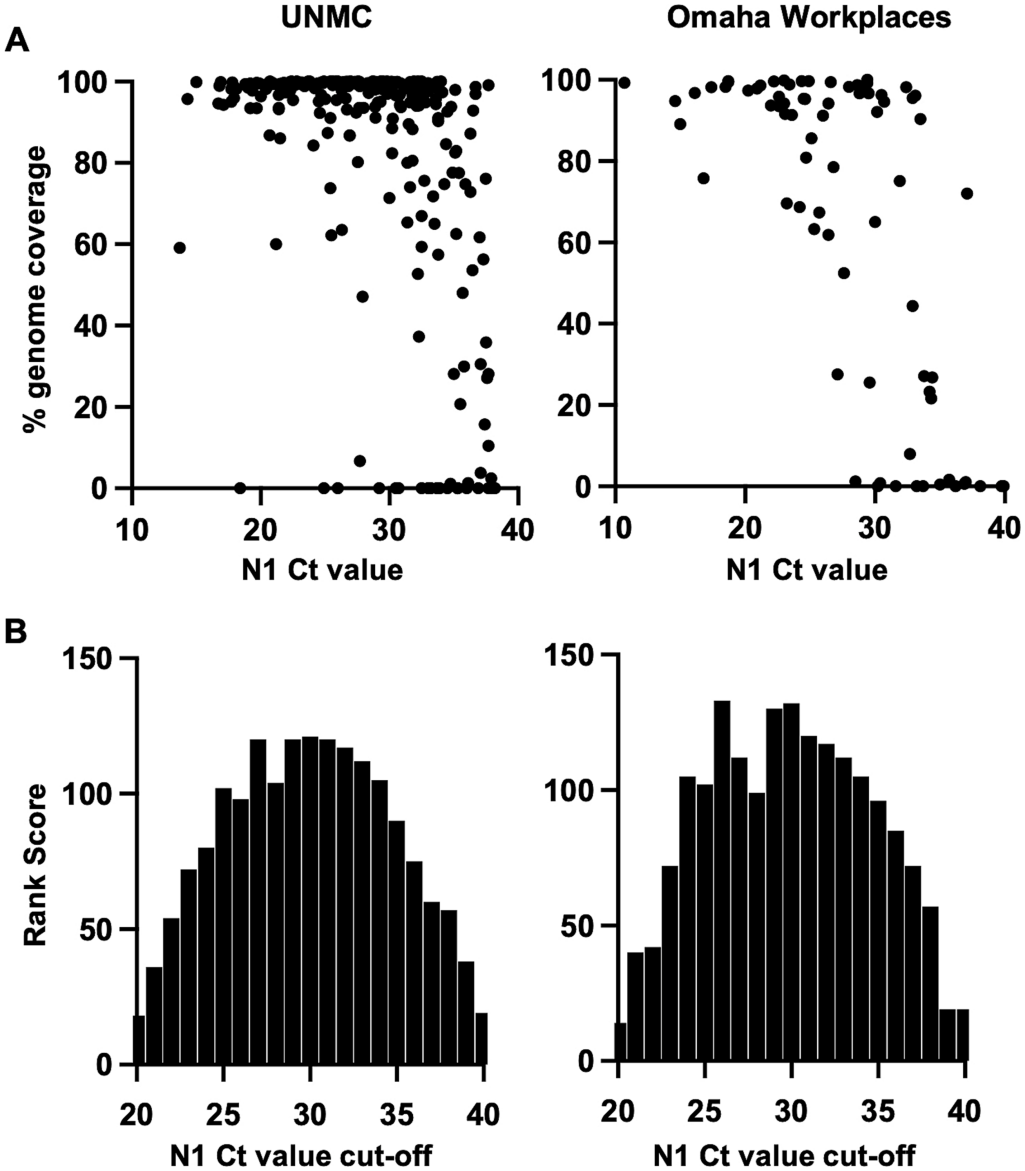
**(A)** SARS-CoV-2 genome coverage achieved by sequencing analysis across N1 cycle threshold (Ct) values of PCR-positive saliva specimens. **(B)** Analysis of optimal N1 Ct value cutoff for sequencing referral of PCR-positive saliva specimens based on the rank score for sensitivity and efficiency of SARS-CoV-2 variant determination.

The ability to generate adequate SARS-CoV-2 genomic sequence coverage for variant determination is dependent on the amount of virus in the specimen, which is inversely related to the Ct value of the virus PCR target. SARS-CoV-2 genome coverage by sequencing analysis showed a robust inverse correlation with N1 target Ct value (Figure 2A). In linear regression analyses of percent genome coverage, including negative natural logarithmic and logarithmic transforms, the relationship with N1 Ct value remained significant when adjusted for location (UNMC, Omaha Workplaces; *p* < 0.0001). Mantel–Haenszel chi square analysis showed a high likelihood of higher percent genome coverage at a N1 Ct value of less than 35 (*p* < 0.0001).

As sequencing analysis is resource intensive, we sought to determine optimal N1 Ct value cutoffs for sequencing referral in our specimen sets. The approach of referring all PCR-positive specimens (i.e., N1 Ct <40) for sequencing yields the highest sensitivity for variant determination. However, the efficiency of sequencing is expected to improve if specimens with a low level of virus (i.e., high Ct value) are excluded. The sensitivity and efficiency of sequencing for SARS-CoV-2 variant determination was calculated across a range of positive N1 Ct value cut-offs from 20 to 40, and rank scores were applied (Supplementary Table S1). The product of rank scores for sensitivity and efficiency (“Rank Score,” Supplementary Table S1) was plotted across N1 Ct cut-off values, demonstrating a similar distribution in both program settings with the highest scores occurring at Ct cut-off values of 26–33 (Figure 2B).

## 4 Discussion

Innovative and sustainable strategies are needed to support community-based SARS-CoV-2 genomic surveillance, particularly in populations with limited healthcare access (23–25). We demonstrate successful integration of SARS-CoV-2 sequencing for variant determination into screening programs in occupational settings using self-collected saliva with an extraction-free qRT-PCR testing method. This approach offers the advantages of a non-invasive specimen collection, no need for supervised collection by a healthcare worker, supply chain resiliency, distributable access, and scalability. High specimen quality across program settings demonstrated the suitability of self-collected saliva specimens for PCR and genomic surveillance testing.

Despite similar distributions of SARS-CoV-2 N1 gene target Ct values, positive specimens collected at the UNMC program site showed a higher rate of successful variant determination by genomic sequencing than those collected off campus and transported to the laboratory. We also observed a lower rate of obtaining ≥99% genome coverage (which is typically required for investigating transmission chains) in specimens collected off campus. This may reflect RNA degradation during transport that, while not impacting amplification of the PCR target, diminishes whole-genome sequencing coverage. Biases inherent in self-selection for testing and readily available testing may have contributed to this finding (e.g., self-perceived risk and testing early in the course of infection). Work is ongoing to evaluate sources of variation in sequence recovery between program settings. Strategies to mitigate RNA degradation during specimen transport include the use of RNA preservative reagents and maintenance of cold chain. However, these strategies add cost and complexity and must be carefully evaluated to determine the added value to testing program quality.

To the best of our knowledge, we present the first generalizable approach to optimize specimen referral for variant determination by sequencing analysis. Applying rank scores to a range of virus gene target Ct value cut-offs for sensitivity and efficiency of variant determination can be used by laboratories to assess their processes employing local data. A universal Ct value cutoff cannot be applied, as the sensitivity of variant determination is dependent on sequencing method and RNA integrity, and efficiency is dependent on viral load (Ct value) distribution in the testing population. During periods of low community transmission, prioritizing sensitivity for variant determination is advisable, whereas efficiency becomes most relevant during periods of high community transmission and resource scarcity.

Each testing program was unique with respect to the participating populations and recruitment design. Consequently, performance differences could be influenced by non-delineated factors. The participant population described in this study was predominantly white adults (majority women for the UNMC campus and majority men for the Omaha workplaces). Factors such as vaccination status, age, and immune states may impact viral load (and therefore Ct values); however, we did not collect vaccination status or store individual demographic or clinical data linked to Ct values. The bulk of observations were among Omicron-lineage SARS-CoV-2 viruses. Findings in programs using extraction-based PCR may differ, reinforcing the importance of pursuing such approaches with local data. Future work will include (1) the application of saliva-based genomic surveillance to additional SARS-CoV-2 variants and other respiratory pathogens including seasonal and avian influenza viruses, (2) implementation of saliva-based genomic surveillance in additional community settings to assess the generalizability of our findings across diverse demographic groups, (3) exploration of pathogen and population characteristics that influence program quality metrics, and (3) optimization of specimen preservation for downstream molecular detection and genomic characterization.

Representation of underserved communities in genomic surveillance of respiratory pathogens can be improved through increased community access to PCR-based testing. Improving the effectiveness and sustainability of sequencing-based variant monitoring relies upon optimizing laboratory-anchored surveillance programming and specimen referral patterns. The utilization of self-collected saliva for respiratory pathogen surveillance and genomic monitoring may facilitate broader community engagement and representation, supporting public health policy and risk communication driven by locally relevant data. This approach may also be implemented for focused surveillance of high-risk groups, such as individuals with occupational exposures to emerging respiratory pathogen threats including avian influenza viruses.

Saliva has been successfully utilized in SARS-CoV-2 screening and surveillance programs in healthcare settings, college campuses, and schools (6, 8–11). Our study further demonstrates successful implementation of saliva-based testing and genomic surveillance in occupational settings during periods of high and low community case rates. However, community-based testing programs using self-collected saliva specimens may face challenges spanning quality assurance, information technology including test registration and reporting, community acceptability, privacy, regulatory compliance, and cost. Additional studies performed in diverse settings and populations should be pursued to further establish successful strategies to meet these challenges. As highlighted by our study, defining quality metrics to track key program outputs is critical for iterative program optimization informed by local data.

## Data Availability

The original contributions presented in the study are included in the article/Supplementary material, further inquiries can be directed to the corresponding authors.

## References

[1] van Dorp L Acman M Richard D Shaw LP Ford CE Ormond L . Emergence of genomic diversity and recurrent mutations in SARS-CoV-2. J Mol Epidemiol Evol Genet Infect Dis. (2020) 83:104351. doi: 10.1016/j.meegid, PMID: 32387564 PMC7199730

[2] Goswami C Sheldon M Bixby C Keddache M Bogdanowicz A Wang Y . Identification of SARS-CoV-2 variants using viral sequencing for the Centers for Disease Control and Prevention genomic surveillance program. BMC Infect Dis. (2022) 22:404. doi: 10.1186/s12879-022-07374-7, PMID: 35468749 PMC9035976

[3] Worobey M Pekar J Larsen BB Nelson MI Hill V Joy JB . The emergence of SARS-CoV-2 in Europe and North America. Science. (2020) 370:564–70. doi: 10.1126/science.abc8169, PMID: 32912998 PMC7810038

[4] Genomic sequencing of SARS-CoV-2: a guide to implementation for maximum impact on public health. (2021). Available online at: https://www.who.int/publications-detail-redirect/9789240018440 (Accessed March 28, 2023).

[5] Gangavarapu K Latif AA Mullen JL Alkuzweny M Hufbauer E Tsueng G . Outbreak.info genomic reports: scalable and dynamic surveillance of SARS-CoV-2 variants and mutations. Nat Methods. (2023) 20:512–22. doi: 10.1038/s41592-023-01769-3, PMID: 36823332 PMC10399614

[6] Wyllie AL Fournier J Casanovas-Massana A Campbell M Tokuyama M Vijayakumar P . Saliva or nasopharyngeal swab specimens for detection of SARS-CoV-2. N Engl J Med. (2020) 383:1283–6. doi: 10.1056/NEJMc2016359, PMID: 32857487 PMC7484747

[7] Rayack EJ Askari HM Zirinsky E Lapidus S Sheikha H Peno C . Routine saliva testing for SARS-CoV-2 in children: methods for partnering with community childcare centers. Front Public Health. (2023) 11:1003158. doi: 10.3389/fpubh.2023.1003158, PMID: 36817891 PMC9936085

[8] Bordi L Sberna G Lalle E Fabeni L Mazzotta V Lanini S . Comparison of SARS-CoV-2 detection in nasopharyngeal swab and saliva samples from patients infected with omicron variant. Int J Mol Sci. (2023) 24:4847. doi: 10.3390/ijms24054847, PMID: 36902277 PMC10003189

[9] Chopoorian A Banada P Reiss R Elson D Desind S Park C . Persistence of SARS-CoV-2 in saliva: implications for late-stage diagnosis and infectious duration. PLoS One. (2023) 18:e0282708. doi: 10.1371/journal.pone.0282708, PMID: 36928472 PMC10019618

[10] Alpert T Vogels CBF Breban MI Petrone ME Wyllie AL Grubaugh ND . Sequencing SARS-CoV-2 genomes from saliva. Virus Evol. (2022) 8:veab098. doi: 10.1093/ve/veab098, PMID: 35542310 PMC9074962

[11] Crowe J Schnaubelt AT SchmidtBonne S Angell K Bai J Eske T . Assessment of a program for SARS-CoV-2 screening and environmental monitoring in an urban public School District. JAMA Netw Open. (2021) 4:e2126447. doi: 10.1001/jamanetworkopen.2021.26447, PMID: 34550382 PMC8459193

[12] Campbell WS Donahue M Williams RM McCutchen E Broadhurst J Schnaubelt A . A public health laboratory information system in support of health emergencies: the Nebraska public health laboratory experience. Public Health Rep. (2023) 138:602–9. doi: 10.1177/00333549231168459, PMID: 37125740 PMC10133861

[13] Ceron JJ Lamy E Martinez-Subiela S Lopez-Jornet P Capela-Silva F Eckersall P . Use of saliva for diagnosis and monitoring the SARS-CoV-2: a general perspective. J Clin Med. (2020) 9:1491. doi: 10.3390/jcm9051491, PMID: 32429101 PMC7290439

[14] Policy for Coronavirus Disease-2019 Tests. (2023). Food and Drug Administration. Available online at: https://www.fda.gov/regulatory-information/search-fda-guidance-documents/policy-coronavirus-disease-2019-tests-revised (Accessed November 15, 2021).

[15] Vogels C Brackney DE Chaney CK Ott IM Grubaugh N Wyllie A. SalivaDirect: RNA extraction-free SARS-CoV-2 diagnostics V.5. (2020). Available online at: https://www.protocols.io/view/salivadirect-rna-extraction-free-sars-cov-2-diagno-bkjgkujw?version_warning=no (Accessed September 1, 2020).

[16] Vogels CBF Watkins AE Harden CA Brackney DE Shafer J Wang J . Yale IMPACT research team. SalivaDirect: a simplified and flexible platform to enhance SARS-CoV-2 testing capacity. Med. (2021) 2:e6:263–80. doi: 10.1016/j.medj.2020.12.010 PMC7836249 33521748

[17] Addetia A Lin MJ Peddu V Roychoudhury P Jerome KR Greninger AL. Sensitive recovery of complete SARS-CoV-2 genomes from clinical samples by use of swift biosciences’ SARS-CoV-2 multiplex amplicon sequencing panel. J Clin Microbiol. (2020) 59:e02226–20. doi: 10.1128/jcm.02226-20, PMID: 33046529 PMC7771467

[18] Greninger Lab Covid Swift Pipepine. (2024). Available online at: https://github.com/greninger-lab/covid_swift_pipeline (Accessed January 15, 2023).

[19] nCoV-2019 sequencing protocol v2 (GunIT) V.2. protocols.io. (2021). Available online at: https://www.protocols.io/view/ncov-2019-sequencing-protocol-v2-bp2l6n26rgqe/v2 (Accessed October 1, 2021).

[20] nCoV-2019 Novel coronavirus bioinformatics protocol. ARTIC Network. (2020). Available online at: https://artic.network/ncov-2019/ncov2019-bioinformatics-sop.html (Accessed October 1, 2021).

[21] Nextclade. Nextstrain. (2024). Available online at: https://clades.nextstrain.org (Accessed October 1, 2021).

[22] COVID Data Tracker. Centers for Disease Control and Prevention. (2023). Available online at: https://covid.cdc.gov/covid-data-tracker (Accessed February 26, 2023).

[23] Ling-Hu T Rios-Guzman E Lorenzo-Redondo R Ozer EA Hultquist JF. Challenges and opportunities for global genomic surveillance strategies in the COVID-19 era. Viruses. (2022) 14:2532. doi: 10.3390/v14112532, PMID: 36423141 PMC9698389

[24] New WHO 10-year strategy aims to scale up genomic surveillance. Pan American health organization. (2022). Available online at: https://www.paho.org/en/news/31-3-2022-new-who-10-year-strategy-aims-scale-genomic-surveillance (Accessed April 3, 2023).

[25] Brito AF Semenova E Dudas G Hassler GW Kalinich CC Kraemer MUG . Global disparities in SARS-CoV-2 genomic surveillance. Nat Commun. (2022) 13:7003. doi: 10.1038/s41467-022-33713-y, PMID: 36385137 PMC9667854

