# Supplementary material for: SARS-CoV-2 genomic surveillance using self-collected saliva specimens during occupational testing programs
Source: Front Public Health. 2025 Apr 8;13:1360862. doi: 10.3389/fpubh.2025.1360862 (PMC12011812; doi:10.3389/fpubh.2025.1360862)
Supplement: Supplementary file 1 [file Data_Sheet_1.docx]

Supplementary Material

# Supplementary Figures and Tables

## Supplementary Figures

**
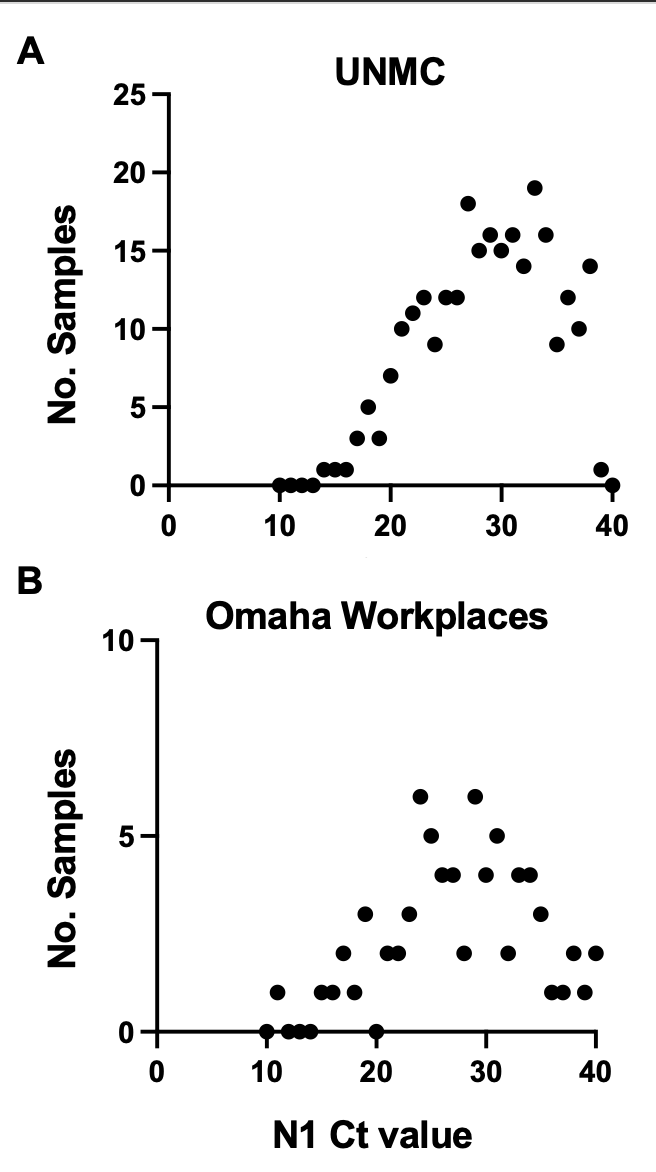
Supplementary Figure 1.** Distribution of N1 Ct values in SARS-CoV-2-positive saliva specimens collected in two program settings, December 2021-November 2022.

**Supplementary Figure 2.** Percentage of SARS-CoV-2 variants detected across program periods and settings, December 2021-November 2022.

**
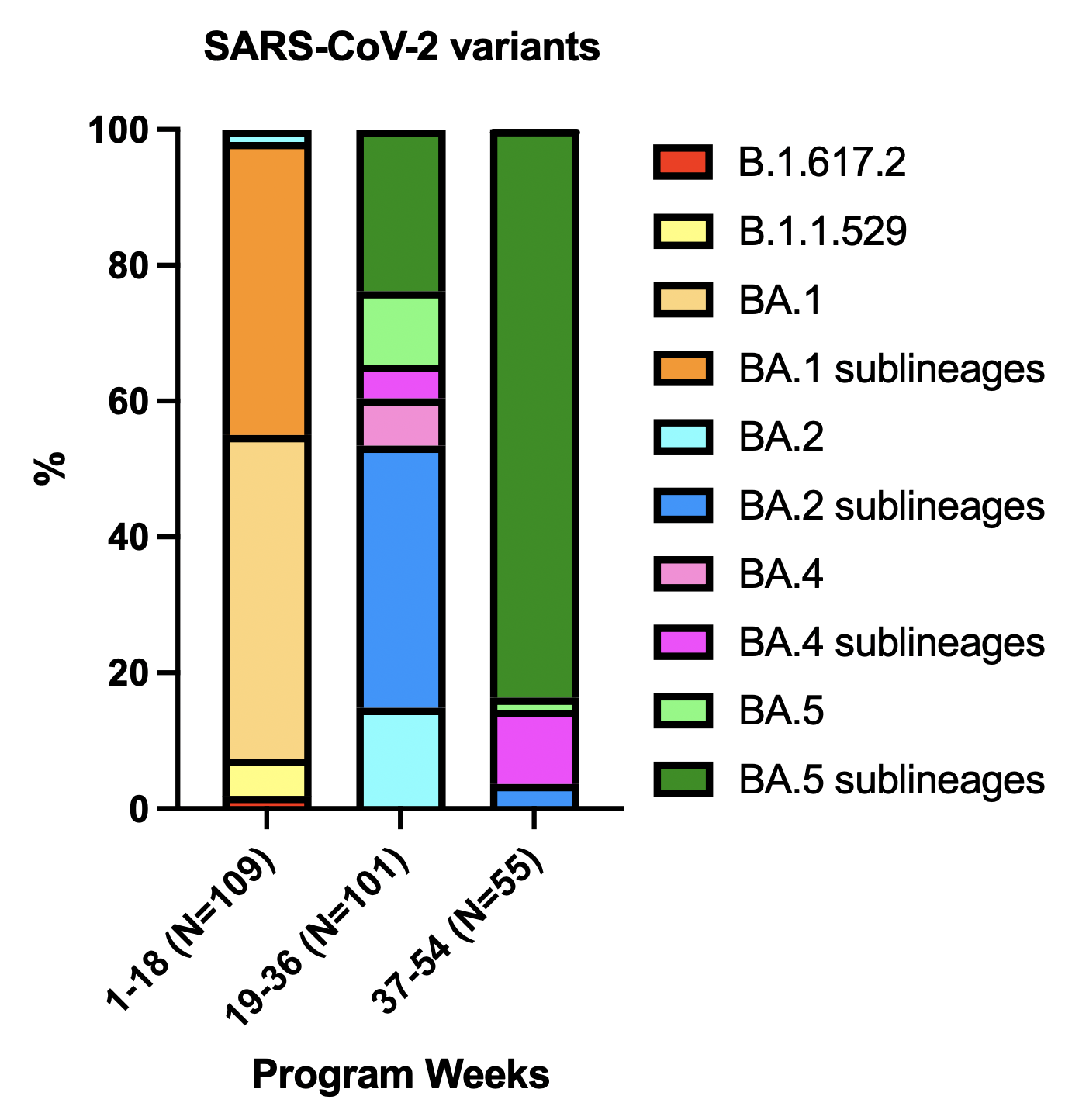
**

## Supplementary Tables

**Table S1.** Calculated sensitivity and efficiency of sequencing for SARS-CoV-2 variant determination across a range of N1 Ct value cut-offs.

| \| Ct cutoff \| No. attempted \| No. successful^a^ \| Efficiency^b^ \| Sensitivity^c^ \| Rank: Efficiency \| Rank: Sensitivity \| Rank score \| \| --- \| --- \| --- \| --- \| --- \| --- \| --- \| --- \| \| UNMC \| \| \| \| \| \| \| \| \| 40 \| 262 \| 217 \| 0.828 \| 1.000 \| 1 \| 19 \| 19 \| \| 39 \| 262 \| 217 \| 0.828 \| 1.000 \| 1 \| 19 \| 19 \| \| 38 \| 261 \| 217 \| 0.831 \| 1.000 \| 3 \| 19 \| 57 \| \| 37 \| 247 \| 215 \| 0.870 \| 0.991 \| 4 \| 18 \| 72 \| \| 36 \| 237 \| 210 \| 0.886 \| 0.968 \| 5 \| 17 \| 85 \| \| 35 \| 225 \| 204 \| 0.907 \| 0.940 \| 6 \| 16 \| 96 \| \| 34 \| 216 \| 197 \| 0.912 \| 0.908 \| 7 \| 15 \| 105 \| \| 33 \| 200 \| 186 \| 0.930 \| 0.857 \| 8 \| 14 \| 112 \| \| 32 \| 181 \| 170 \| 0.939 \| 0.783 \| 9 \| 13 \| 117 \| \| 31 \| 167 \| 157 \| 0.940 \| 0.724 \| 10 \| 12 \| 120 \| \| 30 \| 151 \| 143 \| 0.947 \| 0.659 \| 12 \| 11 \| 132 \| \| 29 \| 136 \| 129 \| 0.949 \| 0.594 \| 13 \| 10 \| 130 \| \| 28 \| 120 \| 113 \| 0.942 \| 0.521 \| 11 \| 9 \| 99 \| \| 27 \| 105 \| 100 \| 0.952 \| 0.461 \| 14 \| 8 \| 112 \| \| 26 \| 87 \| 84 \| 0.966 \| 0.387 \| 19 \| 7 \| 133 \| \| 25 \| 75 \| 72 \| 0.960 \| 0.332 \| 17 \| 6 \| 102 \| \| 24 \| 63 \| 61 \| 0.968 \| 0.281 \| 21 \| 5 \| 105 \| \| 23 \| 54 \| 52 \| 0.963 \| 0.240 \| 18 \| 4 \| 72 \| \| 22 \| 42 \| 40 \| 0.952 \| 0.184 \| 14 \| 3 \| 42 \| \| 21 \| 31 \| 30 \| 0.968 \| 0.138 \| 20 \| 2 \| 40 \| \| 20 \| 21 \| 20 \| 0.952 \| 0.092 \| 14 \| 1 \| 14 \| \| Omaha Workplaces \| \| \| \| \| \| \| \| \| 40 \| 72 \| 48 \| 0.667 \| 1.000 \| 1 \| 19 \| 19 \| \| 39 \| 70 \| 48 \| 0.686 \| 1.000 \| 2 \| 19 \| 38 \| \| 38 \| 69 \| 48 \| 0.696 \| 1.000 \| 3 \| 19 \| 57 \| \| 37 \| 67 \| 47 \| 0.701 \| 0.979 \| 4 \| 15 \| 60 \| \| 36 \| 66 \| 47 \| 0.712 \| 0.979 \| 5 \| 15 \| 75 \| \| 35 \| 65 \| 47 \| 0.723 \| 0.979 \| 6 \| 15 \| 90 \| \| 34 \| 62 \| 47 \| 0.758 \| 0.979 \| 7 \| 15 \| 105 \| \| 33 \| 58 \| 45 \| 0.776 \| 0.938 \| 8 \| 14 \| 112 \| \| 32 \| 54 \| 43 \| 0.796 \| 0.896 \| 9 \| 13 \| 117 \| \| 31 \| 52 \| 42 \| 0.808 \| 0.875 \| 10 \| 12 \| 120 \| \| 30 \| 47 \| 40 \| 0.851 \| 0.833 \| 11 \| 11 \| 121 \| \| 29 \| 43 \| 37 \| 0.860 \| 0.771 \| 12 \| 10 \| 120 \| \| 28 \| 37 \| 32 \| 0.865 \| 0.667 \| 13 \| 8 \| 104 \| \| 27 \| 35 \| 32 \| 0.914 \| 0.667 \| 15 \| 8 \| 120 \| \| 26 \| 31 \| 28 \| 0.903 \| 0.583 \| 14 \| 7 \| 98 \| \| 25 \| 27 \| 26 \| 0.963 \| 0.542 \| 17 \| 6 \| 102 \| \| 24 \| 22 \| 21 \| 0.955 \| 0.438 \| 16 \| 5 \| 80 \| \| 23 \| 16 \| 16 \| 1.000 \| 0.333 \| 18 \| 4 \| 72 \| \| 22 \| 13 \| 13 \| 1.000 \| 0.271 \| 18 \| 3 \| 54 \| \| 21 \| 11 \| 11 \| 1.000 \| 0.229 \| 18 \| 2 \| 36 \| \| 20 \| 9 \| 9 \| 1.000 \| 0.188 \| 18 \| 1 \| 18 \|   ^a^Defined by ability to determine variant strain.  ^b^Efficiency is defined by the fraction of all sequencing attempts that yield a successful variant call when applying the designated Ct value cut-off for sequencing referral.  ^c^Sensitivity is defined by the fraction of all successful variant calls achieved when applying the designated Ct value cut-off for sequencing referral. |
| --- | --- | --- | --- | --- | --- | --- | --- | --- | --- | --- | --- | --- | --- | --- | --- | --- | --- | --- | --- | --- | --- | --- | --- | --- | --- | --- | --- | --- | --- | --- | --- | --- | --- | --- | --- | --- | --- | --- | --- | --- | --- | --- | --- | --- | --- | --- | --- | --- | --- | --- | --- | --- | --- | --- | --- | --- | --- | --- | --- | --- | --- | --- | --- | --- | --- | --- | --- | --- | --- | --- | --- | --- | --- | --- | --- | --- | --- | --- | --- | --- | --- | --- | --- | --- | --- | --- | --- | --- | --- | --- | --- | --- | --- | --- | --- | --- | --- | --- | --- | --- | --- | --- | --- | --- | --- | --- | --- | --- | --- | --- | --- | --- | --- | --- | --- | --- | --- | --- | --- | --- | --- | --- | --- | --- | --- | --- | --- | --- | --- | --- | --- | --- | --- | --- | --- | --- | --- | --- | --- | --- | --- | --- | --- | --- | --- | --- | --- | --- | --- | --- | --- | --- | --- | --- | --- | --- | --- | --- | --- | --- | --- | --- | --- | --- | --- | --- | --- | --- | --- | --- | --- | --- | --- | --- | --- | --- | --- | --- | --- | --- | --- | --- | --- | --- | --- | --- | --- | --- | --- | --- | --- | --- | --- | --- | --- | --- | --- | --- | --- | --- | --- | --- | --- | --- | --- | --- | --- | --- | --- | --- | --- | --- | --- | --- | --- | --- | --- | --- | --- | --- | --- | --- | --- | --- | --- | --- | --- | --- | --- | --- | --- | --- | --- | --- | --- | --- | --- | --- | --- | --- | --- | --- | --- | --- | --- | --- | --- | --- | --- | --- | --- | --- | --- | --- | --- | --- | --- | --- | --- | --- | --- | --- | --- | --- | --- | --- | --- | --- | --- | --- | --- | --- | --- | --- | --- | --- | --- | --- | --- | --- | --- | --- | --- | --- | --- | --- | --- | --- | --- | --- | --- | --- | --- | --- | --- | --- | --- | --- | --- | --- | --- | --- | --- | --- | --- | --- | --- | --- | --- | --- | --- | --- | --- | --- | --- | --- | --- | --- | --- | --- | --- | --- | --- | --- | --- | --- | --- | --- | --- | --- | --- | --- | --- | --- | --- | --- | --- | --- | --- | --- | --- | --- | --- | --- | --- | --- | --- | --- | --- | --- | --- | --- | --- | --- | --- | --- | --- | --- | --- | --- |
